# Dry Eye Analysis: A Citation Network Study

**DOI:** 10.1155/2019/3048740

**Published:** 2019-08-14

**Authors:** Miguel Angel M. A. Sanchez-Tena, Cristina C. Alvarez-Peregrina, Cesar C. Villa-Collar

**Affiliations:** School of Biomedical and Health Sciences, Universidad Europea de Madrid, Madrid 20822, Spain

## Abstract

**Introduction:**

Dry eye is one of the most frequent eye problems with prevalence and incidence from 5% to 50%. Citation network analysis allows us to simplify information in a visual way and provides a better understanding of the research done in a specific field. The objective of this paper is to quantify and analyse the relationships among the scientific literature in this field using citation network analysis.

**Materials and Methods:**

The program used to analyse the citations was CitNetExplorer®. Previously, papers published in the research field during a predefined period were found using the keywords defined in Web of ScienceTM (WOS).

**Results:**

Using the keyword “dry eye,” during the period 2007 to 2018, the most cited paper is by Lemp, MA (2007), with a citation index score of 913 in our citation network containing 6,500 most relevant papers. Analysing clustering, we found 5 relevant groups that match the main areas of research in this field: definition and classification, treatment, retina, refractive surgery, and quality of vision. Core Publication is composed of 64% of the papers in the network, which is a high percentage. It indicates a clear focus on the research carried out in this field.

**Conclusions:**

This citation network analysis shows definition and classification of dry eye to be the most researched area in this field, followed by treatment.

## 1. Introduction

Dry eye is one of the most frequent eye problems reported by adults in eye care practitioner consultations. The prevalence and incidence vary from 5% to 50%, depending on the diagnosis criteria or on the type of dry eye. Women and older people are the most affected. Dry eye prevalence is rising, due to the gradual increase of the population age and to a higher incidence of some risk factors [1].

Dry eye was defined as a disease just 30 years ago. Since then, there have been many advances in the knowledge and definition of this pathology. In 1995, it was defined as a “disorder of the tear film due to tear deficiency or excessive evaporation, which causes damage to the interpalpebral ocular surface and is associated with symptoms of ocular discomfort”. In 2017, a new definition was published in the DEWS II report [2].

Nowadays, according to the abovementioned report, dry eye is defined as “a multifactorial disease of the ocular surface characterized by a loss of homeostasis of the tear film and accompanied by ocular symptoms, in which tear film instability and hyperosmolarity, ocular surface inflammation and damage, and neurosensory abnormalities play etiological roles” [3]. This new definition admits the multifactorial origin of dry eye and points out the etiological factors implied in this disease.

Regarding dry eye classification, traditionally, dry eye was classified into aqueous tear-deficient and evaporative. Now, we know that both exist as a continuum and, as dry eye progresses, it is used to find characteristics of both subtypes [2]. The aqueous tear-deficient dry eye implies a failure of lacrimal tear secretion, while the evaporative dry eye is related to eyelids and ocular surface.

The great number of patients suffering from dry eye and the impact of this disease on patients both financially and in terms of quality of life justify the analysis of all the research studies published about this public health problem.

Citation network analysis allows us to simplify information in a visual way and provides a better understanding of the research done in a specific field. It also lets us quantify the most cited papers and create groups based on connections between papers and citation frequencies [4]. Citation network analysis has been a very useful tool since the citation index concept appeared.

These citation networks appear as different fields related through connections. Authors, journals, and papers are an essential part of these networks [5].

Because research on dry eye pathology is so extensive, use of this citation analysis methodology will allow us to identify the most relevant authors, publications, and journals, as far as their citation is concerned, the most important years in terms of their publications, and the different clusters of study within dry eye pathology.

The aim is to identify not only the most relevant research but also the different areas of study and thus focus future research on dry eye.

The objective of this paper is to quantify and analyse the relationships among the scientific literature in this field using citation network analysis.

## 2. Materials and Methods

CitNetExplorer® was used to analyse the citation networks of individual publications. This software is a tool for visualizing the most important publications in a field and showing the citation relations between these publications [6].

First, the researchers define the keywords and look for the publications according to these words in Web of ScienceTM (WOS). The file from WOS shows papers in the research field published during the predefined period.

The final file is the selection of 6,500 publications of the WOS list sorted by relevance, because the citation network program does not allow more publications to be introduced.

Then, once the file is imported to CitNetExplorer®, the software produces the first graph about the most cited publications, with a maximum of 40 papers for a clearer understanding.

Quantitative analysis shows values of publications, citation links (total number of citations in the network), and time period. This analysis shows the most cited papers in order from the highest to the lowest, according to their citation index score.

The clustering function allows us to identify groups according to the level of association among papers. In this way, subnetworks are obtained depending on the citations among them [7].

Finally, central publications are analysed through the Core Publication function, revealing the main papers of the field. For this analysis, only those that have 4 citations or more are selected.

## 3. Results

The keywords used for the search were “dry eye”. The period chosen was from 2007 to 2018. The year 2007 was chosen as the starting point because of the change that the Dry Eye Workshop (DEWS) report represented in the definition and treatment of dry eye. 9,359 papers were found using the previous criteria.

We made a citation network with 6,500 most relevant papers, obtaining 44,942 citations across the network.

Table 1 shows the 20 most cited papers in this network.

The paper by Lemp et al., published in 2007, is the most cited, with a citation index score of 913.

**Figure 1:**
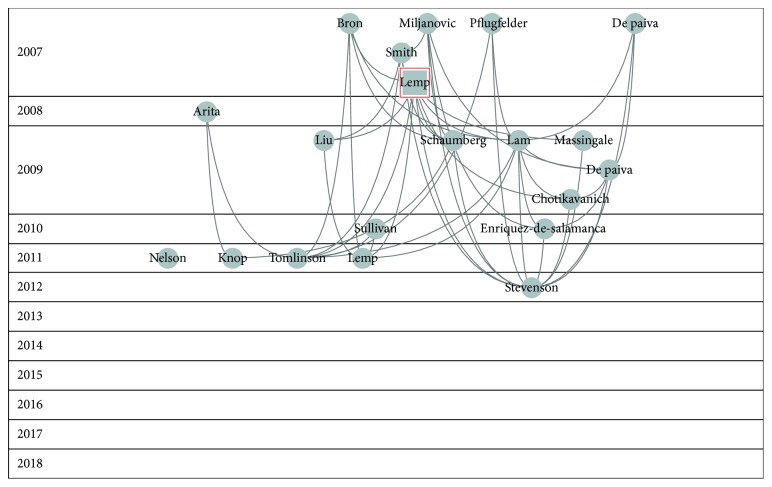
Dry eye citation network graph from CitNetExplorer.

Figure 1 shows the graph of this network.

With the clustering function, we obtained 13 groups or clusters, 5 of them having a relevant number of papers, while the other 8 did not reach 1%.

**Figure 2:**
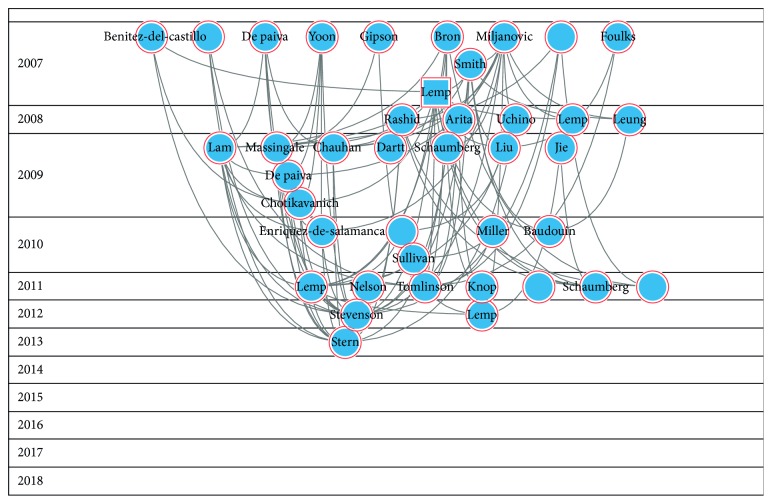
Citation network of cluster 1.

**Figure 3:**
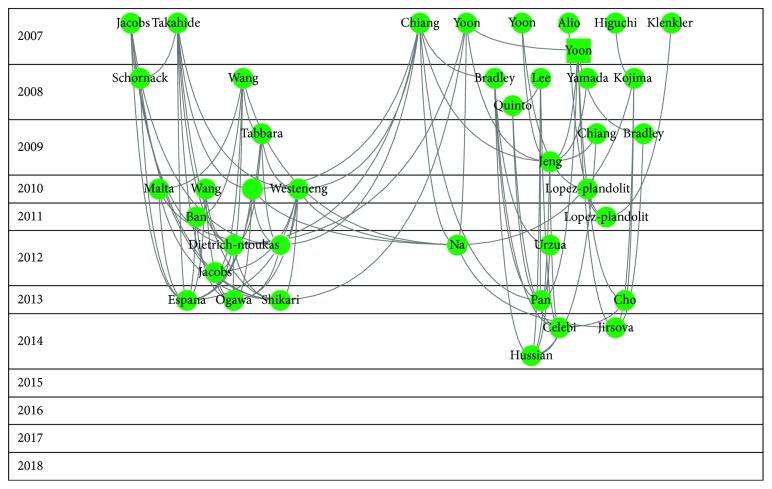
Citation network of cluster 2.

**Figure 4:**
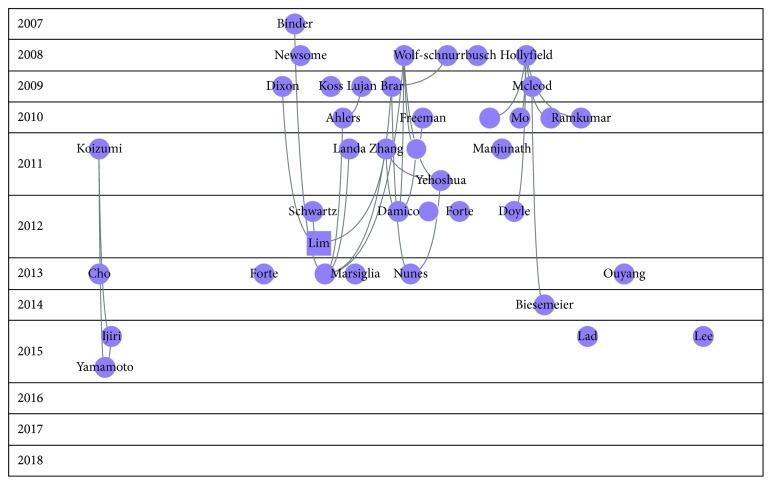
Citation network of cluster 3.

**Figure 5:**
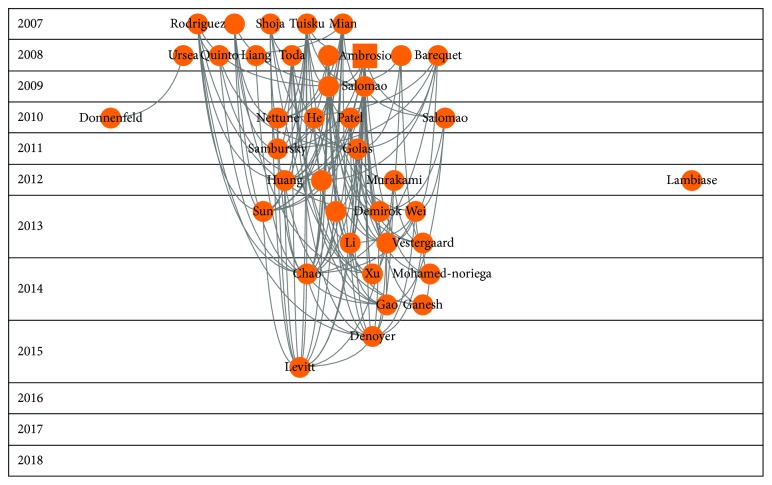
Citation network of cluster 4.

**Figure 6:**
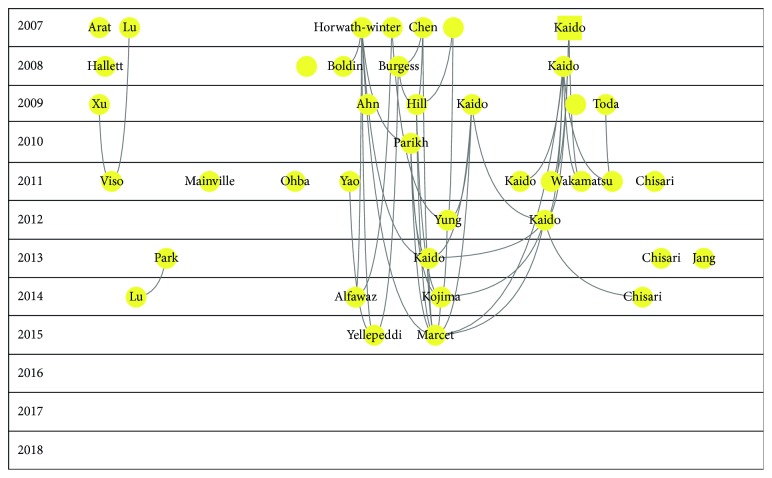
Citation network of cluster 5.

**Figure 7:**
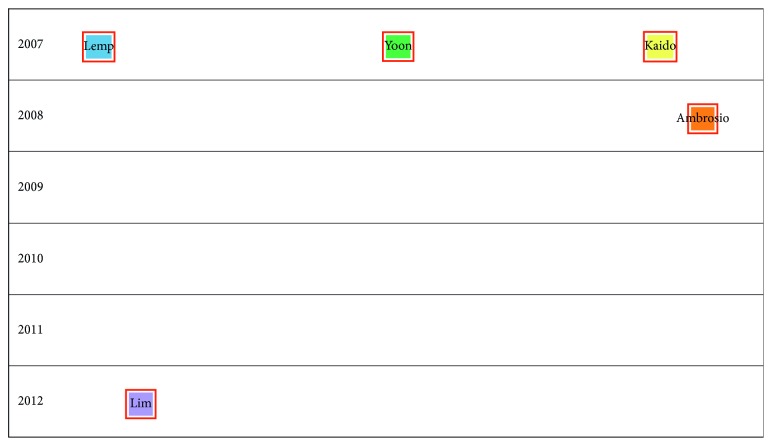
Relationships among different clusters.

Figures 2–6 show the citation network of each group, and Figure 7 shows that there are no citations among different groups.

In group 1, we had 4,014 papers, almost 61% of the network. Lemp's paper, published in 2007 in Ocular Surface Journal [2], was the most cited in this group.

In group 2, we found 360 publications. Yoon's paper, published in 2007 in American Journal of Ophthalmology [26], was the most cited.

In group 3, we found 198 publications. Lim's paper, published in 2012 in The Lancet [27], was the most cited.

In group 4, we found 179 publications. Ambrosio's paper, published in 2008 in Journal of Refractive Surgery [28], was the most cited.

Finally, in group 5, we found 143 publications. Kaido's paper, published in 2007 in Cornea [29], was the most cited.

When we analysed relationships among clusters, we could not find any connections.

**Figure 8:**
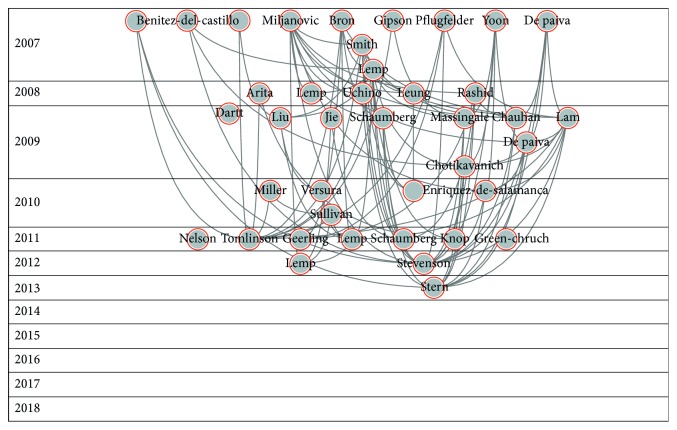
Core Publication of the dry eye citation network analysis.

When we analysed the Core Publication, we found a total of 4,161 papers that cited, or were cited by, at least 4 papers. This 4,161 represented 64% of the papers, and the citation network across this group is 42,791.  Figure 8 shows the graph of this network.

## 4. Discussion

This analysis has proved how publications about dry eye have been increasing in recent years, 2007 being a key year.

There is no doubt about the relevance of the publication of the DEWS report since Lemp et al. in 2007 [2].

The network analysed papers published from 2007 to 2018, but the most cited papers are in the period from 2007 to 2012. This suggests that 2012 could be another key year in dry eye research because of the number and content of papers, analysing the different publications.

Regarding clustering, we found 5 relevant groups that match the main areas of research in this field: definition and classification, treatment, retina, refractive surgery, and quality of vision.

This clustering also shows a difference among the journals that published each of the clusters. It clearly indicates the editorial lines of each scientific journal, no matter how cross the subject was, as in the case of dry eye.

The biggest cluster is the one related to definition and classification of dry eye, with more than 60% of the papers of this network. DEWS report heads this cluster due to the broad consensus on the new definition and classification of dry eye established in this report. This was used until DEWS II was published in July 2017, which included a redefinition of dry eye.

The second cluster we found is related to the treatment. It shows that dry eye is a chronic pathology and how treatment is one of the main challenges for vision professionals. The cluster is led by the prospective case-control study of Yoon et al., published in 2007 in American Journal of Ophthalmology, where autologous serum was compared with umbilical cord serum eye drops in 48 patients with severe dry eye syndrome.

Finding retina as a third cluster could seem surprising because retina has no bearing on the relationship between ocular surface and dry eye. However, it could be explained by the relationship between age and dry eye and between age and pathology of the retina. In other words, studies with older people could shed light on different pathologies such as dry eye and retina problems. It would be worth carrying out further research in the future.

The fourth cluster is about refractive surgery. This is due to the fact that one of the most common complications of LASIK is dry eye. The most cited article in this cluster is a review from Ambrosio et al., published in 2008 in Journal of Refractive Surgery. They reviewed the scientific literature and summarized the experience of the authors to propose methods for decreasing dry eye after surgery.

Finally, the fifth cluster relates to quality of vision. This is one of the concerns for patients suffering from dry eye.

The absence of relationships among the clusters' most cited articles is striking. We assumed we would find some connections between clusters, but in fact, none of the five articles cites any of the others. Therefore, we see five clearly different topics in five differentiated clusters.

Core Publication accounts for 64% of the papers in the network, which is a high percentage. This means there is a clear focus on the research carried out in this field. Definition and classification made up most of this Core Publication. Treatment and refractive surgery are also represented in this core, albeit in less quantity.

## 5. Conclusions

Dry eye is a very important field for researchers, with a very high number of publications and many connections among articles.

This citation network analysis shows that definition and classification of dry eye is still very important, most of the articles being related to this cluster. It is followed in relevance by treatment.

## Figures and Tables

**Table 1:** 20 most cited papers from 2007 to 2018 in the dry eye citation network.

Authors	Paper title	Journal	Year	Citation index
Lemp et al. [2]	The definition and classification of dry eye disease: report of the Definition and Classification Subcommittee of the International Dry Eye Workshop (2007)	The Ocular Surface. 2007 Apr; 5(2): 75–92.	2007	913
Smith et al. [1]	The epidemiology of dry eye disease: report of the Epidemiology Subcommittee of the International Dry Eye Workshop (2007)	The Ocular Surface. 2007 Apr; 5(2): 93–107	2007	380
Miljanović et al. [8]	Impact of dry eye syndrome on vision-related quality of life	American Journal of Ophthalmology. 2007 Mar; 143(3): 409–15.	2007	271
Bron [9]	Methodologies to diagnose and monitor dry eye disease: report of the Diagnostic Methodology Subcommittee of the International Dry Eye Workshop (2007)	The Ocular Surface. 2007 Apr; 5(2): 108–52.	2007	253
Sullivan et al. [10]	An objective approach to dry eye disease severity	Investigative Opthalmology & Visual Science. 2010 Dec; 51(12): 6125–30.	2010	213
Lemp et al. [11]	Tear osmolarity in the diagnosis and management of dry eye disease	American Journal of Ophthalmology. 2011 May; 151(5): 792–798.e1.	2011	211
Schaumberg et al. [12]	Prevalence of dry eye disease among US men: estimates from the Physicians' Health Studies.	Archives of Ophthalmology. 2009 Jun; 127(6): 763–8.	2009	207
Lam et al. [13]	Tear cytokine profiles in dysfunctional tear syndrome.	American Journal of Ophthalmology. 2009 Feb; 147(2): 198–205.	2009	192
Pflugfelder et al. [14]	Management and therapy of dry eye disease: report of the Management and Therapy Subcommittee of the International Dry Eye Workshop (2007)	The Ocular Surface. 2007 Apr; 5(2): 163–78.	2007	182
Knop et al. [15]	The international workshop on meibomian gland dysfunction: report of the Subcommittee on Anatomy, Physiology, and Pathophysiology of the Meibomian Gland.	Investigative Opthalmology & Visual Science. 2011 Mar 30; 52(4): 1938–78.	2011	171
Stevenson et al. [16]	Dry eye disease: an immune-mediated ocular surface disorder.	Archives of Ophthalmology. 2012 Jan; 130(1): 90–100.	2012	156
Massingale et al. [17]	Analysis of inflammatory cytokines in the tears of dry eye patients.	Cornea. 2009 Oct; 28(9): 1023–7.	2009	147
De Paiva et al. [18]	IL-17 disrupts corneal barrier following desiccating stress.	Mucosal Immunology. 2009 May; 2(3): 243–53.	2009	146
Nelson et al. [19]	The international workshop on meibomian gland dysfunction: report of the Definition and Classification Subcommittee	Investigative Opthalmology & Visual Science. 2011 Mar 30; 52(4): 1930–7.	2011	141
Tomlinson et al. [20]	The international workshop on meibomian gland dysfunction: report of the Diagnosis Subcommittee.	Investigative Opthalmology & Visual Science. 2011 Mar 30; 52(4): 2006–49.	2011	133
Liu et al. [21]	A link between tear instability and hyperosmolarity in dry eye.	Investigative Opthalmology & Visual Science. 2009 Aug; 50(8): 3671–9.	2009	129
Arita et al. [22]	Noncontact infrared meibography to document age-related changes of the meibomian glands in a normal population.	Ophthalmology. 2008 May; 115(5): 911–5.	2008	128
De Paiva et al. [23]	Dry eye-induced conjunctival epithelial squamous metaplasia is modulated by interferon-gamma.	Investigative Opthalmology & Visual Science. 2007 Jun; 48(6): 2553–60.	2007	122
Enríquez de Salamanca et al. [24]	Tear cytokine and chemokine analysis and clinical correlations in evaporative-type dry eye disease.	Molecular Vision. 2010 May 19; 16: 862–73.	2010	122
Chotikavanich et al. [25]	Production and activity of matrix metalloproteinase-9 on the ocular surface increase in dysfunctional tear syndrome.	Investigative Opthalmology & Visual Science. 2009 Jul; 50(7): 3203–9.	2009	120

## Data Availability

The data used to support the findings of this study are available from the corresponding author upon request.
